# Indicators for the sustainability assessment of MBR technologies for wastewater reuse in Chile: The good, the bad, and the ugly

**DOI:** 10.1016/j.mex.2023.102111

**Published:** 2023-03-06

**Authors:** Montserrat Rodríguez-Castillo, Vanessa Bolívar-Paypay, Witold-Roger Poganietz, Ana Lucía Prieto

**Affiliations:** a Departamento de Ingeniería Civil, Universidad de Chile, Av. Blanco Encalada 2002, Santiago, Chile; b Centro Avanzado para Tecnologías del Agua (CAPTA), Universidad de Chile, Av. Beauchef 850, Santiago, Chile; c Institute for Technology Assessment and Systems Analysis, Karlsruhe Institute of Technology, Germany

**Keywords:** Membrane bioreactors, Resource recovery, Sustainability indicators, Wastewater reuse, Life cycle assessment, ICoS, Sustainability framework, Advanced wastewater treatment, Integrative Concept of Sustainable Development (ICoS)

## Abstract

While Chile faces a mega-drought, wastewater reuse emerges as an alternative solution. In this study we develop a set of indicators for the comprehensive sustainability assessment for the application of advanced wastewater treatment technologies (e.g., MBRs) in a wastewater reuse project in Chile. The methodology is based on the Integrative Concept of Sustainable Development (ICoS) framework. A critical analysis of the set of indicators is presented in terms of the benefits (The Good), the difficulties (The Bad), and the barriers (the Ugly) for their development and potential application. The characterization of the environmental benefits constitutes the useful aspects (e.g., recovery of nutrients, energy, and water). Difficulties include economic aspects (e.g., continuous monitoring of emerging contaminants) and public acceptance. Political and administrative aspects were found to be the main barrier, including water rights in Chile and the absence of a clear regulatory framework for wastewater reuse. To our knowledge, this study is the first to present a detailed methodology for developing indicators for membrane-based water reuse projects in Chile.

The steps to develop the indicators are:

• Identification of the study zone or case study, characterization of treatment technology.
• Identification and formulation of indicators for the specific case study, based on the ICoS framework.
• Verification of the relevance of indicators for the case study according to data availability and expert reviews.

## Graphical abstract

**Figure:**
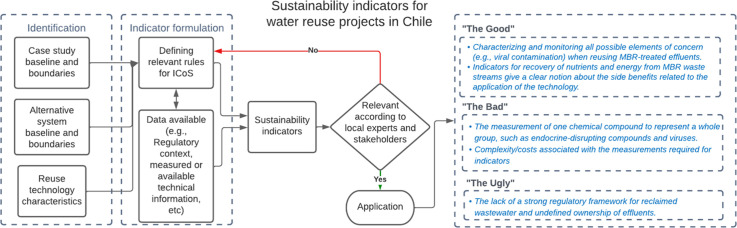

Specifications table

**Table:** 

Subject area:	Environmental Science
More specific subject area:	Sustainability in wastewater reuse
Name of your method:	Integrative Concept of Sustainable Development (ICoS)
Name and reference of original method:	Kopfmüller, J., Brandl, V., Jörissen, J., Paetau, M., Banse, G., Coenen, R. & Grunwald, A. (2001). Nachhaltige Entwicklung integrativ betrachtet. Konstitutive Elemente, Regeln, Indikatoren. Berlín: Taschenbuch.
Resource availability:	N/A

## Introduction

Since 2010, Chile has been in a prolonged mega-drought [1], which has led the local government to declare “water scarcity zones” in several regions of the country [2]. As Chile strives to maintain the current supply and meet the growing demand, reusing treated wastewater is arising as a viable alternative. Currently, there are 301 wastewater treatment plants (WWTPs) throughout the country, treating approximately 1.216 billion m 3 of wastewater in 2020 (average flow of 40.7 m 3 /s) [3]. As for the final destination of treated municipal wastewater, 96% is discharged back into inland surfaces or marine water bodies, of which 13% (8.8 m 3 /s) is discharged into the sea through submarine outfalls/emissaries after preliminary treatment. Except for the submarine emissaries, most of the treated effluents in Chile have assigned water rights downstream from the discharge point, making reuse projects legally challenging [4]. On the other hand, the current Chilean regulatory framework for wastewater reuse projects is still in its infancy. Table 1 summarizes the nationally available policies and regulatory guidelines. Besides the NCh1333 (created in 1978), regulatory guidelines for intended wastewater reuse are quite recent, non-mandatory, and focus on water reuse for non-potable uses. Although the NCh1333 sets water quality requirements for different uses (including irrigation), it does not specify the water source. More recent guidelines (since 2020) do focus on wastewater as a source for reuse applications. For example, guideline NCh3456 (2021) establishes guidelines for developing and implementing projects that intend to use treated wastewater for the irrigation of crops in public and private gardens, considering climate, soil, and water quality parameters.

**Table 1:** Chilean regulatory framework for municipal wastewater reuse.

Regulation	Description
NCh1333 (1978)	Guideline for water quality criteria according to the intended use. Uses include irrigation, animal and human consumption, recreational and esthetic uses, and preservation of aquatic life. Does not specify the source of water.
DS90 (2000)	Supreme Decree regulates the discharge of pollutants into marine and inland surface watercourses by setting maximum permissible limits for liquid waste discharge.
Ley 21.075 (2018)	Law regulates the collection, reuse, and disposal of greywater. Applicable to urban and rural areas for irrigation, toilet flushing, recreational and industrial uses, and maintenance of wetlands.
NCh3465 (2020)	Guidelines for the safety assessment (water quality) and public acceptance parameters for the reuse of treated wastewater and treated greywater in urban areas.
NCh3456/1 (2021)	Guidelines for developing and implementing projects that intend to use treated wastewater for irrigation of crops in public and private gardens, considering climate, soil, and water quality parameters.
NCh3456/2 (2021)	Guidelines to prevent risks to the population having direct or indirect contact with treated wastewater or any product that came into contact with treated wastewater.
NCh3483 (2021)	Guidelines for the classification of water quality based on the type of non-potable water reuse application and the level of exposure of users. In addition, it recommends public disclaimers of the water reuse application (e.g., signs or banners).
NCh3462 (2021)	Guidelines for a centralized water reuse system in two parts:
	– Part 1 provides guidelines for the planning and designing centralized urban water reuse systems applicable to the different components of the water recycling system: source, treatment, storage, distribution, operation, and monitoring.
	– Part 2 refers to principles and methodologies for management in a centralized water reuse system, describing elements related to management issues and recommending actions for incidents, emergencies, and monitoring.

Currently, available technology for wastewater treatment is designed to comply with other regulatory standards that do not consider reuse applications [5]. More advanced technologies should be adopted to provide the highest levels of treatment and contaminant removal that accounts for emerging pollutants such as antibiotics, hormones, persistent organics, etc. Membrane bioreactors (MBR) are an evolving technology globally applied to wastewater reuse. By coupling biological treatment with a membrane filtration unit, MBRs have gained importance over traditional technologies due to their compact nature that removes up to 99% of trace organics and emerging pollutants [6], and its capacity to produce recycled water with high-quality characteristics that can be used for multi-reuse purposes [7], [8], [9], [10].

Although MBRs are commercially available technologies, demonstration sites are needed to substantiate their safe implementation for direct reuse in Chile. Additionally, the sustainability of wastewater reuse projects in the country is still not well understood. Existing research has evaluated the sustainability of water reuse projects from the Life Cycle Assessments (LCA) perspective. Rodriguez et al. (2021) analyzed the sustainability of a graywater reuse project using LCA applied to a treatment system that included activated carbon and zeolite as filtration media [11]. Rodríguez-Merchan et al. (2021) evaluated the water-energy nexus by comparing urban potable water treatment systems in Chile through exergy analysis, LCA, and water-related indicators (i.e., water stress index and blue water footprint) [12]. Vergara et al. 2020) analyzed the environmental impacts of implementing an integrated water, waste, and energy management system in a medium-sized but rapidly growing settlement in a coastal area (Curauma, Chile) [13]. Literature regarding comprehensive sustainability assessment methodologies and their application for wastewater reuse projects in Chile is quite limited. Many other relevant aspects must be considered within the Chilean context, such as different industrial activities and water consumption patterns, lifestyles and cultural customs, geophysical and climate conditions, and national and global political frameworks [14].

This paper describes the methodology to identify critical indicators for the sustainability of a wastewater reuse project in the country through the application of advanced treatment systems such as the MBR. Using a hypothetical implementation site located in the Valparaisoregion of Chile, the MBR plant will produce clean water for local irrigation, among other water needs. Based on the indicators developed according to the Integrative Concept of Sustainable Development (ICoS) framework, we identify advantages or positive/useful aspects (the good), difficult but achievable challenges (the bad), and barriers (the ugly) to the sustainability assessment. The applicability of this system as a water reclamation technology, as well as barriers to its implementation, are discussed in this study.

## Method details

### Overview

The method consists of three steps:

1. Identification of the study zone or case study (i.e., Botanical Gardens of Viña del Mar), characterization of treatment technology (e.g., MBR, treatment train, water sources and demands, and system boundaries). This step defines the conditions for the baseline/current and alternative systems used for comparison during the sustainability assessment.
2. Identification and formulation of indicators for the specific case study, based on the Integrative Concept of Sustainable Development (ICoS) framework. Aspects such as the Chilean regulatory framework and water quality requirements for reuse should be considered when evaluating the indicator's relevance.
3. Verification of the relevance of indicators for the case study according to data availability and expert reviews. Based on interviews with local experts in the different technical, economic, social, and environmental fields, it will be determined the applicability of the indicator to assess the sustainability of the technology in the local context.

#### Case study: botanical gardens of Viña del Mar - Valparaíso Region

The case study will focus on an MBR plant located in the Botanical Gardens of the city of Viña del Mar (BGVM), one of the most important tourist sites both in the city and in the Valparaisoregion. It comprises a man-made lagoon (the LinneoLagoon), which regulates flows of a stream to the BGVM water system and serves as a tourist attraction. The water system receives sporadic storm runoff from the adjacent watershed and from roads that discharge into the creek. The stream water is pumped to a storage tank to be used for irrigation, toilet flushing, and other park services. Due to the regional ongoing drought, the creek's flow has decreased over the years. As of December 2022, the BGVM has 7 L/s of water rights assigned from the BGVM's stream, which barely covers its current water demand without affecting the creek's environmental flow. The BGVM also counts on an additional 13.5 L/s of assigned aquifer water rights that remain unused. Potable water is provided by the local sanitary authority.

Additional inflows into the BGVM include a wastewater sewer main that runs through the garden with an average of 146 L/s of primary treated effluent (i.e., submarine emissary “2 Norte”). Per interviews with the BGVM administrative officer, the BGVM demands approximately 200 m^3^ per month of drinking water to be used in the administration buildings and handwashing stations throughout the BGVM. Latrines connected to septic tanks are distributed throughout the gardens to provide toilet services for visitors. Cleaning services for septic tanks are outsourced, and wastewater is sent to the city of Quillota's WWTP, where it is treated and discharged into the Aconcagua River. Also, irrigation is the main water demand of the BGVM. On average, 1 L/s is used for one hectare of landscape and another 3 L/s are consumed in 32 hectares of forest. These amount to an average daily water demand of 4 L/s, five days a week, all year. Water is also used to fill pools throughout the park that contain endemic species of fish.

For the sustainability assessment, only wastewater from the sewer main and latrine wastewater are considered as the influent to the MBR plant. Using a design flow of 1000 m^3^/d (11.5 L/s), the plant's treatment train consists of primary sedimentation and equalization tanks, followed by an MBR and a chlorine disinfection unit. For sludge management, drying beds are used and the resulting biosolids are applied on-site for soil amendment. Also, coarse solids, oils and fats that resulted after the initial primary sedimentation will be sent to the WWTP in Quillota. As per local regulations, the effluent quality complies with DS 90/2000 standards and the more recently established guidelines for treated wastewater reuse in irrigation, considering contact with humans. The treated effluent will be directly used within the botanical garden. Thus, resources associated with water transport are not considered.

**Fig. 1:**
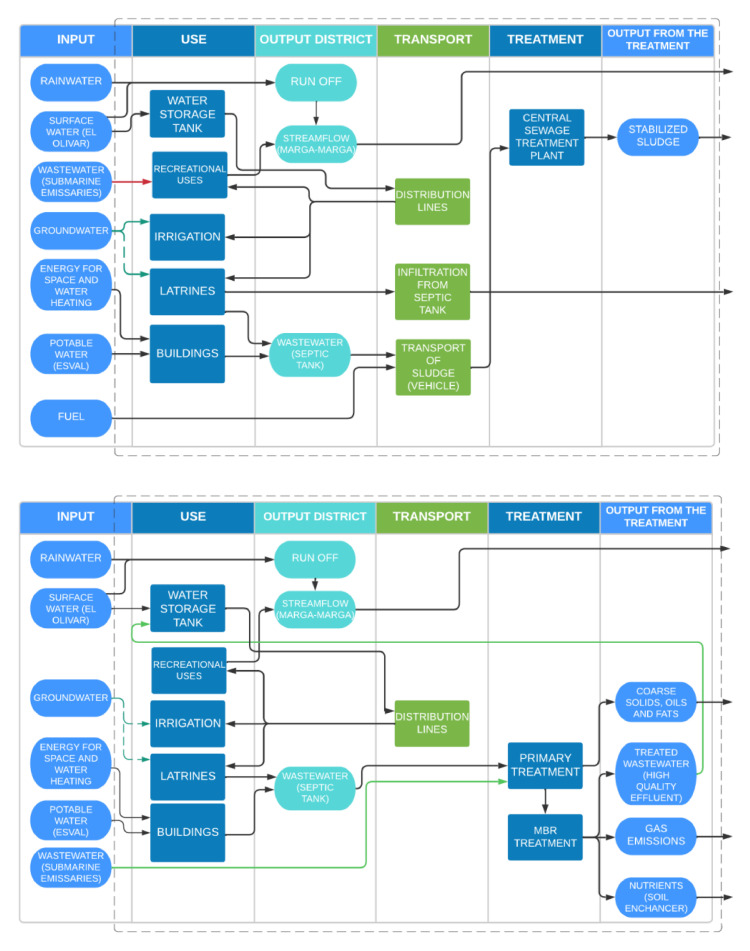
Top: baseline energy and material flow diagram for the BGVM, Bottom: alternative energy and material flow diagram considering the impact of the MBR in the system. Squares represent processes and circles represent materials. Note the groundwater is available but not used due to cost constraints (baseline: dotted green line).

To understand the impact of the MBR technology on the water budget of the BGVM, we organize and identify the relevant processes and flows in the system. Fig. 1 presents the material and energy flow for the BGVM case study. For the baseline system scenario, there are five inputs of water into the BGVM: 1) El Olivar Creek, which supplies water for ground irrigation, latrines, and recreational uses (e.g., LinneoLagoon); 2) rainwater that is transported through El Olivar Creek as surface runoff; 3) potable water that is used for drinking purposes, hand washing, and administrative building needs; 4) groundwater (green dotted arrow in Fig. 1.a), which is currently not used due to the high cost of operation, as the water must be raised (pumped) 30 m from the groundwater level; and 5) wastewater (red arrow in Fig. 1.a) when the submarine emissary overflows. Pipe ruptures and heavy rainfall events generate sporadic inputs of sewer into Olivar Creek, thus polluting the LinneoLagoon. For the purpose of this analysis, it is assumed that irrigation runoff evaporates and does not infiltrate. Flows 1) and 2) gather in the LinneoLagoon to further flow as the lower Olivar Creek into the Marga-Marga River, which crosses the city of Viña del Mar and discharges into the sea.

Other relevant inputs into the system include energy (i.e., electricity) required for space and water heating, and fuel required by the trucks providing latrine cleaning services.

The alternative system scenario includes the treatment of wastewater by an MBR to generate high-quality effluent water for irrigation and recreational purposes. The MBRs improve the quality and quantity of the creek's stream flow, reducing risks associated with overflows and contamination events. It is important to note that the MBR will not only treat the water from the submarine emissary “2 Norte”, but also the wastewater generated in the BGVM. Thus, outputs due to latrine waste collection are eliminated. The sludge from the MBR process will be managed onsite, recovering nutrients in wastewater as biosolids for soil amendment. Thus, an additional use/process is created within the system boundaries. As any other treatment process, the MBR produces emissions into the air (odors and greenhouse gasses), contributing to the carbon footprint of the system.

### Indicators for the sustainability assessment (SA) for MBR treatment and reuse in Chile

#### Integrative concept of sustainable development (ICoS)

Developed by Kopfmüller et al. (2001), the Integrative Concept of Sustainable Development (ICoS) is a framework commonly used to evaluate the sustainability of (new) technologies and systems using a multidimensional approach (technical, economic, environmental, social, and institutional etc.) and the interaction between these dimensions The approach projects three general sustainability goals across all dimensions and translates them into ``rules'' to guide action. [15].

The ICoS framework is based on three goals of sustainability and five sustainability rules for each. These goals focus on: (1) securing human existence, (2) maintaining society's productive potential (comprising natural, man-made, human, and knowledge capital), and (3) preserving society's options for development and action. Each goal has five associated rules, which are selected and translated into different indicators for the specific project in mind. Not every rule proposed by ICoS is addressed for the formulation of the indicators, as they depend upon the context. In our case, a set of indicators for each sustainability goal was formulated based on intensive literature research and local expert interviews (see Tables 1 to 3), which aim to assess the sustainability of implementing an MBR treating municipal wastewater for reuse applications in the BGVM, mainly for irrigation purposes.

To establish the indicators together with the ICoS framework, the following aspects are considered based on the characterization of the case study:

• Water supply and demand: Conduct a comprehensive analysis of water availability and demand in the targeted area to understand the water requirements of different uses, including irrigation, recreational, and for latrines. Determine the sources of water supply and identify potential water reuse opportunities.
• Regulatory framework: Evaluate the regulatory framework for water reuse in Chile to ensure that the project complies with national, regional, and local regulations.
• Water quality requirements: Determine the water quality requirements for different reuse applications, such as irrigation or industrial use. Consider the impact of reuse on the environment and public health.

#### Goal 1: securing human existence

Indicators for the protection of human health aim to guarantee the safe usage of treated wastewater with the selected technology. Due to the high-quality effluents resulting from MBR treatment, it was considered that the safety of the effluent can be assessed not only by quantifying bacteria (indicator 1a), but viruses and contaminants of emerging concern commonly present in municipal wastewater such as endocrine disruptors (EDCs) (Ind. 1b and 1c). These contaminants are widely monitored in conventional wastewater treatment systems, especially considering uses that involve contact with humans. These indicators are extended to the biosolids generated during treatment (Ind. 1d and 1e), since many of the EDCs can accumulate in biosolids and potentially leach into the ground [5]. Emissions and odors are other parameters to be considered for any wastewater treatment technology, since the presence of gasses such as H_2_S could be toxic at high concentrations, might generate undesirable odors for visitors and BGVM's neighbors, and might contribute to greenhouse gas (GHG) emissions (Ind. 1f). Finally, using reclaimed water for landscape irrigation and other purposes (e.g., returned to Olivar Creek) facilitates the availability of blue-green spaces for recreational use and other environmental benefits, including decreasing the heat island effect (Ind. 1 g).

For ensuring the satisfaction of basic needs, the set indicators focus on the resource recovery potential of the MBR process. For instance, the potential to recover nutrients, water, and energy can be assessed by identifying the annual mass of nutrients present in the treated effluent and/or biosolids (Ind. 2a and 2b), the number of alternative sources and annual volume of water generated by the MBR process (Ind. 2c and 2d), and the number of alternative sources of energy and annual quantifiable generation from the MBR (Ind. 2e and 2f).

To assess the just distribution of opportunities for using natural resources in the implementation of the MBR process at the BGVM, a more global indicator should be considered. In this case, the potential emission of the MBR process translated to annual life cycle CO_2eq_ will determine the impact of the process in comparison to the absence of it (Ind. 3a). Table 2 summarizes the indicators for Goal 1.

**Table 2:** Indicators for Goal 1 - Securing human existence.

Rule	Indicator	Unit
1 - Protection of human health	1a - Pathogenic load of bacteria in local streams 1b - Pathogenic load of virus in local streams 1c - Hygienic loads to EDCs in local water streams	E. coli: CFU / 100 ml Coliphage ≥ log 5 reduction; ≤ 5/100 ml (monthly geometric mean) Estradiol: [ng/L]
	1d - Hygienic load to biosolids: pathogens	E. coli: CFU / 100 mg
	1e - Hygienic loads to biosolids: EDCs	Estradiol: [mg/kg]
	1f - Emissions and odors	H_2_S and/or CH_4_ [%v/v], [ppm], or ouE/m^3^
	1g - Provision of urban blue-green spaces with treated wastewater	m^3^ / a of available treated wastewater
2- Ensuring satisfaction of basic needs	2a - Recovered nutrients in water	tons/a of P, N or K, or mass of nutrients in effluent
	2b - Recovered nutrients in biosolids	tons/a of P, N or K, or mass of nutrients in solids
	2c - Diversification of water provision	Number of types of water sources
	2d - Provision of water by alternative sources	m^3^ / a
	2e - Alternative energy sources	Number of types of energy sources
	2f - Provision of renewable energy sources	MWh / a
3 - Just distribution of opportunities for using natural resources	3a - Global warming potential	t CO_2eq_ / a

#### Goal 2: maintaining society's productive potential

Maintaining society's productive potential requires a sustainable use of current resources in order to support future demands. It is important to identify how much water, energy, nutrients, and other renewable or non-renewable resources will be required for the implementation of the MBR technology at the BGVM. In terms of the “sustainable use of renewable resources”, we identified indicators for the BGVMs current drinking water demand and how this compares to future demand as a result of the additional water provided by the MBR (Ind. 4a). Indicator 4b, potential provisions of renewable energy, refers to the current contribution of renewables sources to the Chilean energy mix and additional energy contributions by recovering/converting chemical energy in wastewater.

Regarding non-renewable resources, indicators identified demands of finite resources over the entire life cycle. For the BGVM and MBR process, we considered, for example, fossil fuel demand for transportation (e.g., trucks cleaning the latrines) and indirect fossil fuel consumption via electricity demand. Indicator 5a accounts for the net energy demand of the process. Other resources include the nutrients demanded for BGVM's ground maintenance (Ind. 5b).

For safe wastewater reclamation, assessing the potential risks to the environment is key for the sustainable implementation of any technology. Indicators in the “sustainable use of the environment as a sink” rule include direct and indirect entry of contaminants of emerging concern in local streams, accounting for micropollutants potentially present in the MBR effluent (Ind. 6a, same units as 1c) and those that might leach from the process’ biosolids (Ind. 6b). The amount of biosolids generated from the BGVM by the MBR process that need to be managed or disposed of are accounted for with indicator 6c. The effect of the MBR process emissions is accounted for with the global warming potential indicator (Ind. 6d), eutrophication (Ind. 6e), and acidification potential (Ind. 6f) for each indicator over the entire life cycle.

An MBR reclamation plant at the BGVM provides evidence of efforts in the advancement of wastewater reuse in Chile. Public knowledge of this type of demonstration site should be identified with Indicator 7a.

A cost analysis should provide information regarding the economic feasibility of the desired technology. It should consider construction, operation, and maintenance costs and other relevant parameters that might be used for comparison to alternative technologies/scenarios. With the exception of Ind. 6a., indicators in Table 3 are obtained by life cycle assessment (LCA).

**Table 3:** Indicators for Goal 2 - Maintaining society's productive potential.

Rule	Indicator	Unit
4 - Sustainable use of renewable resources	4a - Drinking water requirements	m^3^ / a
	4b - Provision of renewable energy	MWh / a
5 - Sustainable use of non-renewable resources	5a - Net demand for fossil fuels	t oil_eq_
	5b - Net demand for nutrients by BGVM	t / a of P, N or K, or mass of nutrients in fertilizer
6 - Sustainable use of the environment as a sink	6a - Hygienic loads of EDC to local streams	Estradiol: ng/L in the effluent
	6b - Indirect entry of organic micropollutants in local streams	Target compound [ug / L]
	6c - Solid waste generation by MBR	t waste / year
	6d - Global warming potential	t CO_2eq_ / a
	6e - Eutrophication potential in water bodies	kg P_eq_ / a
	6f - Acidification potential	kg SO_2_ / a
7 - Sustainable development of man-made, human and knowledge capital	7a - Contribution to the people's knowledge with respect to water and energy challenges	Qualified statement
	7b - Construction, operating, and maintenance costs	$ / m^3^

#### Goal 3: preserving society's options for development and action

Availability of reclaimed wastewater can maintain and improve the current environmental conditions of the BGVM. However, as visitors to the BGVM will have direct contact with the irrigated grounds, the LinneoLagoon, and the fauna attracted to the existing water bodies, the acceptance of the reclaimed water is crucial for implementing the MBR technology. If the experience with the recycled wastewater is positive or, at least not negative, the interaction could initiate public acceptance of reclaimed wastewater as an alternative water source in Chile (Ind. 8a). Whereas the reaction of the visitors at BGVM could be rather easily detected, e.g., by the development of the number of visitors or number and intensity of complaints, the broader impact depends on other factors, which are beyond the scope of the study. Thus, with respect to the broader impacts, only the principal acceptability can be disclosed. Another effect of the interaction accompanied by additional information about the technology could be a better understanding of possible technical alternatives to reduce the impacts of drought, increasing the individual social responsibility (Ind. 8b). Like before, only a general willingness can be identified, but not whether the potentially higher responsibility will emerge in day-to-day behavior.

As part of their mission, the BGVM provides a habitat for a number of endemic species. Reclaimed water from the MBR might help to preserve and improve this natural habitat (Ind. 9a) (Table 4).

## Discussion: critical analysis to assess the sustainability of a wastewater reuse project in Chile

In the following sections we discuss the advantages or useful aspects (the good), the difficulties and challenges (the bad), and absolute barriers (the ugly) to the application of the rules and indicators established in Tables 1 to 3, for the use of ICoS to assess this problem.

## The good

Advanced wastewater treatment technologies such as the MBR provide better opportunities for resource recovery in different scenarios. In the case of the BGVM, the MBR treatment train guarantees the availability of a high-quality effluent to be used in diverse applications, including landscape irrigation, streamflow augmentation, and toilet flushing. Acting as an absolute barrier for solids removal, ultrafiltration membranes (pore size < 30 nm) are often applied in municipal wastewater treatment to remove harmful contaminants such as bacteria, viruses, and even emerging contaminants [16]. Thus, monitoring these contaminants (Ind. 1a to 1e, and 6a) is key to guaranteeing the safe use of the treated effluent and biosolids produced. Considering the biosolids from the MBR might be used for soil amendment within the BGVM, the set indicators of bacterial and emerging contaminant loads can promote safe application of the biosolids in situ and the sustainability of new technologies such as the MBR process. Additionally, the indirect contributions to local streams of contaminants leached from biosolids could be identified with Ind. 6b.

Monitoring the presence of toxic and odorous gases (i.e., H_2_S) and environmentally harmful emissions (i.e., CO_2_ and CH_4_) is also key for the safe implementation of MBR technology (Ind. 1f). The presence of odors is often monitored in wastewater treatment plants because they directly affect the neighboring population, and thus the people's perception of the safety of the treatment technology [17]. Methods for their detection are commercially available and user friendly (e.g., electronic nose). Ind. 1f provides valuable information to the sustainability of the reuse project.

Recovering nutrients and energy from the MBR waste streams is also an important advantage to the implementation and sustainability of the technology. Depending on the MBR operational conditions, nutrients can be recovered in the effluent to be used for fertigation of the BGVM grounds (Ind. 2a). The creation of alternative water sources (Ind. 2c and 2d) that do not compete with existing water rights and might overcome existing and future water demands (Ind. 4a) can increase the resilience of the BGVM in the face of the ongoing drought. Biosolids can be used for soil amendment (Ind. 2b), as well as feedstock for energy generation via pyrolysis or combustion (Ind. 2e, 2f and 4b) [18]. However, the final use or disposal of the MBR products are subjected to local legislation. Also, the impact of the conversion process should be accounted for in the sustainability assessment.

With the exception of 1 g, indicators 1a to 2b are motivated by existing national and international regulatory standards (e.g., NCh3456 and US-EPA 2012 Guidelines for Water Reuse), facilitating the identification of potential uses and risks associated with the treated effluent and other products.

Other benefits associated with environmental and social welfare are identified, further supporting the sustainability of new reclamation technologies. For example, availability of additional sources of water for irrigation has environmental benefits (reduces heat island effect) and promotes recreational uses by increasing blue-green areas and maintaining existing lagoons and ponds (Ind. 1 g). Other indicators associated with environmental health and public perception include the system's eutrophication and acidification potential (Ind. 6e and 6f). Maintaining a healthy and aesthetically pleasing ecosystem for touristic sites such as the BGVM is key to its financial functioning.

Regarding public acceptance and/or promotion of societal responsibility (Ind. 8a and 8b), exposing the public to a safe and functional demonstration of water reclamation, located outside of their living environment, provides the opportunity for them to generate informed opinions regarding wastewater reuse. In the particular case of the BGVM, public acceptance could be assessed by quantifying the change in the number of visitors to the park after the implementation of the MBR process. Comments and complaints will also provide information on public acceptance and social responsibility. The BGVM is also responsible for promoting the associated benefits of reclaimed water. This might be easier to achieve since the park already provides environmental education through outreach programs.

## The bad

Even though treated wastewater constitutes an alternative and renewable source of water, taking it to the highest quality standards might be a real challenge. The characteristics of municipal wastewater are inherently variable and influenced by seasonal and environmental conditions, population served, societal behavior, etc. Thus, the indicators considered to guarantee human health (Table 2) might not be sufficient to characterize the totality of contaminants and potentially harmful compounds present in wastewater. For example, Ind. 1d (concentration of estradiol) was selected to represent a series of emerging compounds (i.e., endocrine disruptors) that might share similar physicochemical properties. Contrary to many contaminants of concern that might be harmful to humans and bioaccumulate in nature (e.g., PAHs), estradiol can be efficiently removed with the MBR treatment process [19]. Indicator 1b informs of the presence of pathogens in the effluent after membrane treatment; however, the detection methods for coliphages in wastewater are complex, require specialized laboratories (e.g., PCR analysis), and might not be economically feasible for small and/or decentralized projects. Other indicators that require data from laboratory work are 2a and 2b, where elemental nutrients (i.e., N, K and P) in the effluent and biosolids should be quantified.

**Table 4:** Indicators for Goal 3 - Preserving society′s options for development and action.

Rule	Indicator	Unit
8 - Conservation of social resources (tolerance, solidarity, etc.)	8a - Acceptance by BGVM visitors	Qualified statement
	8b - Promotion of societal responsibility	Qualified statement
9 - Conservation of the cultural function of nature	9a - Preserved endemic species	Number of species / a

Regarding the indicator “emissions and odors” (Ind. 1f), measuring odors’ nuisance caused by the operation of the MBR plant will vary depending on the distance from the monitoring stations to the plant and the meteorological conditions, among other parameters [20]. Ind. 1f may be expensive if other gasses besides CO_2_, CH_4_ and H_2_S are considered for a deeper analysis. Therefore, a standardized methodology for monitoring odors will be important to assess the impact of the MBR system in the BGVM. In Chile, even though there are guidelines to measure odors from wastewater treatment plants (WWTPs), these are not mandatory [21].

An important consideration for the sustainability of reuse projects in Chile is associated with the economic costs, i.e., construction, operation, and maintenance costs, Ind. 7b), which can significantly influence decision makers with respect to the execution of reuse projects [22]. When implementing new technologies for reuse, the estimated overall costs might be higher than the ones associated with conventional treatment (e.g., CAS), especially considering many of these technologies are developed elsewhere and “imported” to cover the growing demand of local reuse markets. In addition, new and/or redundant infrastructure (e.g., reclaimed and potable distribution lines), materials (e.g., ultrafiltration membranes), and highly skilled labor are required while implementing advanced treatment projects. However, high skilled labor would improve the quality of the regional human resources. Furthermore, some indicators are influenced by the existence of adequate infrastructure. For example, indicators related to new/alternative energy sources from the MBR plant (i.e., 2e, 2f, and 4b) would depend on the existence of waste-to-energy infrastructure at the BGVM (e.g., pyrolysis unit) or an existing market for the biosolids produced. Otherwise, energy recovery may be challenging, as conversion processes may be too costly for on-site implementation. The techno-economic feasibility of reuse projects could outweigh other associated benefits such as environmental welfare and social justice [23].

As some of the indicators are obtained from an LCA, this might be limited by the lack of life cycle inventories (LCIs) and life cycle impact assessments (LCIA) developed specifically for Chile and have therefore relied on international databases that do not accurately reflect the local context [24]. Data availability is an important constraint for indicators such as 3a, 5a, 5b, 6b to 6f. Other limitations to the selected indicators are those inherent to LCA, which include setting the system boundary, setting a proper functional unit, and describing the material fluxes that describe the impact wastewater reuse project as accurately as possible [25].

Finally, public acceptance plays a key role in the sustainability of any reuse project in Chile. For instance, a 2019 citizens’ survey showed 93% of the interviewees think wastewater is a viable resource to alleviate the national water scarcity. Nevertheless, only 22% trust that the reclaimed water is up to the right quality standards to cover drinking water demands [26]. Increasing public acceptance (Ind. 8a,) will greatly depend on people's familiarity with the reuse applications [27]. Many actors are involved in this. At BGVM, it will depend not only on the visitors’ willingness to participate in the park's activities, but the BGVM's promotion of their water conservation practices (Ind. 7a). The employees should then be instructed or kept informed of the plant's general operation and associated benefits. This might generate additional workloads to the BGVM staff already responsible for the park's operation.

Regarding the potential use of treated wastewater for drinking purposes (Ind. 4a), there is no clear regulation in Chile on which institution will control this application (e.g., the ministry of health or the superintendency of sanitary services). Also, the users of the BGVM are expected to collaborate in accepting knowledge and information regarding the why of the use of reclaimed water and its importance. Ind. 7a depends heavily on the willingness of BGVM visitors and users to be informed and support these changes and implement behavioral adjustments when needed. Changes in these framework conditions can easily lead to social controversy. Due to the events of the spread of cholera in the early 1990s or of norovirus in the north of the country due to irrigation with raw or untreated wastewater [28,29], there might still be a collective memory of the harm caused by the use of this water in productive activities.

## The ugly

The safe application of reclaimed water is a continuous topic of discussion in Chile. There are several considerations that might impact the implementation and further sustainability of wastewater reuse projects. First, there is still legal uncertainty as to who owns the wastewater in the submarine emissaries. Even though wastewater is an alternative resource to overcome the effects of drought in the region, conflicting water rights can be an impediment to any reuse project. As the wastewater reuse market/policy in Chile is still in its infancy, an indicator to assess the legal aspects that might impact reuse projects was not included at this point.

As for the regulations for water reuse in Chile, available resources for compliance might be limited or non-existent. For example, we have established that monitoring emerging contaminants is key for the safe implementation of reuse projects. The testing and monitoring of these compounds is not always feasible due to the costs and complexity associated with the experimental methods for their detection in the water and biosolids (Ind. 1c and 1e) [7]. National regulatory guidelines for effluent discharge/reuse do not consider contaminants of emerging concern (e.g., the supreme decree DS90/2000 and NCh3456), thus international regulations and recommendations should be followed. We selected estradiol as the measuring unit for Ind. 1c and 1e, as it is one of the few EDCs considered in international water reuse guidelines (i.e., no effect concentration at less than 0.35 ng/L) and has a direct effect on human and environmental health (e.g., fish feminization) [18]. Viruses (Ind. 1b), on the other hand, are currently not regulated in Chile. Coliphages were chosen as the measuring unit of viruses, as they are indicators of fecal contamination [30], which is relevant for our case study. As the measurement of these indicators needs extensive laboratory processing, it may result in high costs for the BGVM to assume, considering that sampling is not mandatory. In consequence, the above-mentioned indicators might be disregarded from the sustainability analysis, thus not covering all the desired aspects for the sustainability of technology and its application.

Finally, economic subsidies for reclaimed water projects are scarce in Chile. Without subsidies, community-based or non-industry related projects, these kinds of projects may be infeasible because the investment and operation and maintenance costs might not be compensated by water savings or other environmental benefits [22].

## Conclusions

The present analysis delved into the strengths, difficulties, and challenges of carrying out a sustainability analysis for a wastewater reuse project in the Botanical Gardens of Viña de Mar under the specifics of Chile. It was concluded that a sustainability assessment of MBR technology in Chile based on a complex set of indicators, selected and categorized following the ICoS approach, it is in general terms feasible for the study case in BGVM. Nevertheless, there are important considerations to take into account while conducting the sustainability assessment. For instance, the advantages and useful aspects (the good) of the selected set of indicators focus on characterizing and monitoring all possible elements of concern (e.g., viral contamination) when reusing MBR-treated effluents. Also, indicators that measure the recovery of nutrients and energy from MBR waste streams give a clear notion about the side benefits related to the application of the technology. These indicators are motivated by existing quality standards and quantification/measuring protocols. Since the BGVM promotes nature conservation and provides green space areas for local/tourists in the Valparaíso region, a clear understanding of the health and environmental impacts of the treated wastewater is required. The use of indicators helps to monitor the performance of the water reuse system and evaluate its impact on the environment and public health. Also, helps to continuously assess the economic and social benefits of the project and make any necessary adjustments to the reuse plan and management practices.

Indicators identified with difficulties (the bad) were predominantly associated with the availability of information (e.g., LCA input data), and uncertainty regarding economic and political developments that influence the decision-making process for water reuse projects in Chile (e.g., energy-intensive and high operational costs). Other important problems for the implementation of MBRs in the local context include the perception of and the complexity/costs associated with the measurements required for indicators in the “securing human existence” goal. For instance, using one chemical compound to characterize a whole group as in the case of EDCs and viruses is a problem. If all compounds of interest were measured, the BGVM would incur higher costs that might not be affordable or sustainable over time. Concerning the absolute barriers (the ugly) to the sustainability assessment, the absence of a robust regulatory framework for reclaimed wastewater and undefined ownership of effluent in the submarine outfalls are a key impediment to reuse projects in Chile.

## CRediT authorship contribution statement

**Montserrat Rodríguez-Castillo:** Conceptualization, Investigation, Writing – original draft. **Vanessa Bolívar-Paypay:** Writing – review & editing. **Witold-Roger Poganietz:** Writing – review & editing. **Ana Lucía Prieto:** Conceptualization, Methodology, Supervision, Funding acquisition, Writing – review & editing.

## Declaration of Competing Interest

The authors declare that they have no known competing financial interests or personal relationships that could have appeared to influence the work reported in this paper.

## Data Availability

No data was used for the research described in the article.
